# Plasma Photoinactivation of Bacterial Isolated from Blood Donors Skin: Potential of Security Barrier in Transfusional Therapy

**DOI:** 10.3390/pathogens13070577

**Published:** 2024-07-11

**Authors:** Yanet Ventura-Enríquez, Antonio Casas-Guerrero, María de Jesús Sánchez-Guzmán, Miguel Ángel Loyola-Cruz, Clemente Cruz-Cruz, Andres Emmanuel Nolasco-Rojas, Emilio Mariano Durán-Manuel, Dulce Milagros Razo Blanco-Hernández, Francisco Álvarez-Mora, Gabriela Ibáñez-Cervantes, Mónica Alethia Cureño-Díaz, Juan Manuel Bello-López, Verónica Fernández-Sánchez

**Affiliations:** 1 Banco de Sangre, Centro Médico Naval (CEMENAV), Mexico City 04470, Mexico; 2 Hospital Juárez de México, Mexico City 07760, Mexico; 3 Escuela Superior de Medicina, Instituto Politécnico Nacional, Mexico City 11340, Mexico; 4 Facultad de Estudios Superiores Iztacala (FES-Iztacala), Universidad Nacional Autónoma de México (UNAM), Mexico City 54090, Mexico

**Keywords:** bacterial inactivation, skin bacteria, plasma, blood components, riboflavin, UV light

## Abstract

The presence of skin bacteria capable of forming biofilm, exhibiting antibiotic resistance, and displaying virulence represents a significant challenge in the field of transfusion medicine. This underscores the necessity of enhancing the microbiological safety of blood and blood components against pathogens with virulent characteristics. The aim of this work was to demonstrate bacterial inactivation in plasma by using a photoinactivation method against virulent bacteria and to evaluate coagulation factors before and after treatment. Logarithmic loads of biofilm-producing, antibiotic-resistant, and virulent bacteria isolated from skin (*Enterobacter cloacae*, *Klebsiella ozaenae*, and *Staphylococcus epidermidis*) were used in artificial contamination assays of fresh frozen plasma bags and subjected to photoreduction. FVIII and FI activity were evaluated before and after photoinactivation. The photoinactivation of plasma was demonstrated to be an effective method for the elimination of these bacteria. However, the efficiency of this method was found to be dependent on the bacterial load and the type of test microorganism. Conversely, decay of coagulation factors was observed with net residual activities of 61 and 69% for FVIII and FI, respectively. The photoinactivation system could have a bias in its effectiveness that is dependent on the test pathogen. These findings highlight the importance of employing technologies that increase the safety of the recipient of blood and/or blood components, especially against virulent bacteria, and show the relevance of the role of photoinactivation systems as an option in transfusion practice.

## 1. Introduction

One of the most significant challenges currently facing blood banks is the transfusion of safe blood and blood components. The SARS-CoV-2 pandemic has highlighted the necessity of expanding the diversity of infectious markers to enhance recipient safety. This is particularly relevant given the active circulation of the SARS-CoV-2 virus in asymptomatic donors of COVID-19 [1,2]. In contrast, recent waves of migration from South America and Africa to North American countries have posed another risk as a source of importation of endemic pathogens to other countries. Previous studies have demonstrated the importation of malaria, a blood-borne parasitic disease, into Mexico [3,4]. This phenomenon shows the problem of the possible transmission of *Plasmodium* spp. species since these pathogens are not screened in blood donors according to the Mexican Official Standard NOM-253-SSA1-2012, “for the disposal of human blood and its components for therapeutic purposes” [5]. The limitation in blood screening has resulted in the transmission of parasitic diseases through blood transfusion and its components [6,7]. Conversely, molecular tests such as nucleic acid tests (NATs) have been added to revolutionize screening by allowing the detection of donors in the window period [8,9].

Nevertheless, even when technologies have gathered their efforts in serological and molecular screening of blood, there are steps in the donation protocols that have been devalued and have a negative impact as potential sources of contamination, the insufficient antisepsis of the donors’ skin [10]. The skin microbiota is constituted by a robust and complex cellular architecture, where skin structures and skin-inhabiting bacteria are involved [11]. Most of the bacteria are found in the form of biofilms, and therefore, antisepsis procedures become relevant within the donation protocols, as they are difficult to eliminate. Being polymicrobial biological structures, biofilms are composed of bacteria of various genera and species, including fungi and yeasts, and are intimately related to the layers of the skin (stratum corneum, epidermis, dermis, and basal glands). They can be a source of contamination in the collection system [12]. This is why other types of technologies, such as the inactivation of pathogens, become relevant as additional barriers to the safety of the recipient. According to the NOM-253-SSA1-2012, inactivation consists of subjecting a blood component to in vitro treatment with the aim of preventing the transmission of infectious agents, graft-versus-host disease, and other pathologies. Among these technologies are photodynamic, photochemical, solvent/detergent, or other inactivation methods that allow the maintenance of therapeutic properties (viability and non-toxicity in the recipient). Some have shown greater safety for the recipient since they do not involve toxic compounds, and in terms of cost, they are more accessible [13]. The use of photochemical methods (INTERCEPT^TM^ and MIRASOL^®^) employing nucleic acid intercalating molecules that are compatible with the receptor are oriented to the inactivation of pathogens in platelet concentrates and plasma [14,15].

These methodologies, despite their initial focus on the inactivation of pathogens such as hepatitis B and C viruses, HIV, and *Trypanosoma cruzi*, among others, have also been demonstrated to be effective in vitro against skin commensal bacteria [16]. Nonetheless, in other trials that have demonstrated the effectiveness of photochemical methods on bacterial models, they have considered bacteria validated for their ability to proliferate in blood components [17] and have omitted relevant aspects that could influence the photochemical activity of pathogen inactivation systems, such as resistance to chemical and physical agents by the ability to form mature biofilms, antimicrobial resistance, and virulence. Under this premise, in a previous study by our working group, we were able to isolate and identify bacteria from the skin of blood donors that were shown to be intrinsically resistant to povidone iodine (10%) and chlorhexidine gluconate/isopropyl alcohol (2%/70%) (in the form of biofilm), antimicrobial-resistant, and exoenzyme-producing. In this work, we speculate that resistance to povidone and chlorhexidine gluconate/isopropyl alcohol could be associated with the presence of phenotypes and genotypes of mature biofilm formation on living and inert surfaces, and we discuss the role of this virulence factor in resistance to the action of blood bank antiseptics.

Therefore, it is relevant to have information that supports the effectiveness of photoinactivation methods with microbial models that are endowed with physical and chemical protection against various biocidal agents. The aim of this study was to demonstrate ex vivo bacterial inactivation in plasma bags by using a photochemical method and to evaluate their procoagulant properties by detecting the activity of FVIII (antihemophilic factor A) and FI (fibrinogen) before and after photochemical treatment. Implications on the potential risk of acquiring transfusion-transmitted infections of blood components contaminated by resistant and virulent bacteria and the need to increase blood safety in the recipient are analyzed and discussed.

## 2. Materials and Methods

### 2.1. Bacterial Strains and Growth Conditions

Bacterial strains used in this study were provided by Sánchez-Guzmán et al. (2024) from Hospital Juárez de México (HJM) and are listed in Table 1 [10]. All strains were genetically identified by *16S rRNA* gene sequence analysis and characterized to form strong mature biofilms on polystyrene (by violet crystal method), antimicrobial resistance (by CLSI, 2023), and exoenzyme production (in solid phase). Strains were grown in Luria-Bertani broth (LB) (10 g/L tryptone, 5 g/L yeast extract, and 5 g/L NaCl) or LB-agar (supplemented with 1.5% agar) [18]. Finally, the strains were maintained in planktonic form in isotonic saline solution before being used in photoinactivation assays.

### 2.2. Preparation of Bacterial Planktonic Inoculums

Bacterial strains were tawed and streaked on LB-agar and incubated at 37 °C overnight. One colony of each strain was inoculated in 5 mL of LB-broth under agitation at 200 rpm at 37 °C overnight. The planktonic cultures were appropriately diluted in cold isotonic saline solution and were spread (per triplicate) onto LB-agar plates and incubated overnight for the CFU/mL counting. With the calculated CFU values, appropriate adjustments were made to contain 10^5^, 10^6^, and 10^7^ CFU/mL. Bacterial suspensions were stored and refrigerated at 4 °C for 18 h before use on an ice bed.

### 2.3. Collection of Fresh Frozen Plasma Bags

All blood donations in this work were from eligible, non-remunerated voluntary donors from HJM. Twenty-seven bags of fresh plasma containing 197.2 ± 22.4 mL were obtained after fractionation of whole blood by using the REVEOS^®^ system (Terumo Blood and Cell Technologies, Lakewood, CO, USA). For this purpose, collection bags (Teruflex^®^ Terumo Hatagaya, Shibuya, Japan; containing CPDA-1 solution) were used. Twenty-seven units of whole blood (480 ± 20 mL) from donors were obtained. Fractionation of the blood units was within the first six hours post-donation and immediately frozen at −30 °C, considering these bags as fresh frozen plasma. Plasmas bags were maintained at this temperature until use for bacterial inactivation assays (three months). All bags were tested for infectious serology of the blood bank according to Mexican regulation (antibodies against *Trypanosoma cruzi*, *Brucella, Treponema pallidum*, HIV, HVB, and HCV and nucleic acid testing (NAT)) before experimentation.

### 2.4. Bacterial Contamination, FVIII/FI Determination, and Photoinactivation

**Figure 1:**
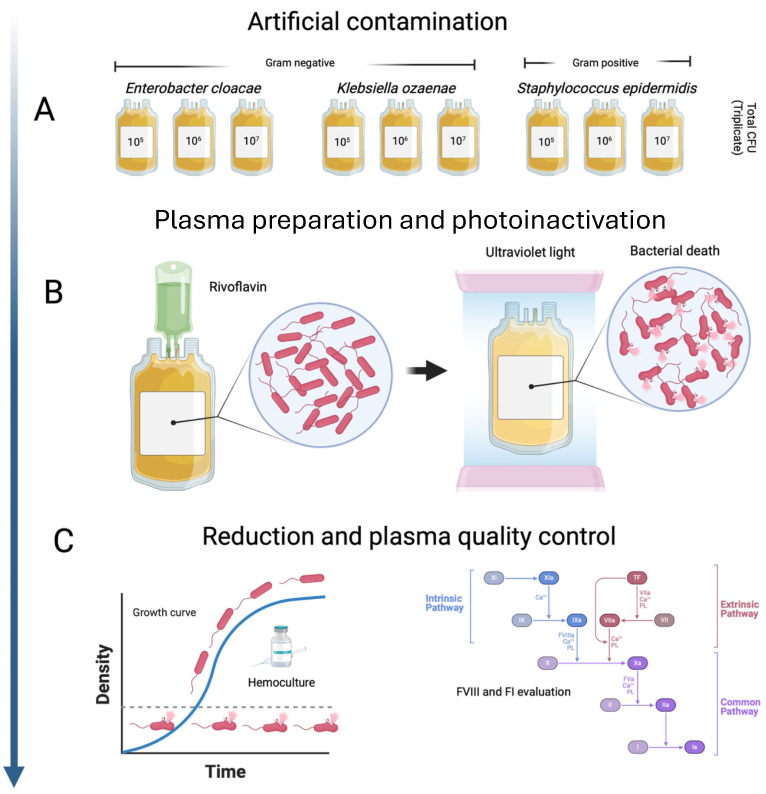
General working scheme of the photoinactivation of artificially contaminated plasma bags with bacteria isolated from skin. (**A**) Artificial contamination with skin bacteria, (**B**) Plasma bags preparation and photoinactivation and (**C**) Reduction and plasma quality control.

Plasma bags were thawed at a controlled temperature (37 °C) in a water bath (Barkey plasmaTherm, Cambridge, MA, USA) for 20 min and perfectly mixed. FVIII activation FI quantification and artificial contamination assays were carried out under aseptic conditions in a type 2 laminar flow cabinet. Before artificial contamination, 1 mL of plasma was aseptically removed from each bag with a sterile syringe prior to sanitization of the sampling site bag with ethanol (70%, *v*/*v*) for activation and quantification of FVIII and FI, respectively. The above measures were determined in duplicate by using a STAR Max3 automatic coagulation analyzer (Diagnostica Stago, Asnières-sur-Seine, France). Bacterial suspensions of each strain were inoculated into plasma bags, as indicated below. Immediately, one milliliter of inoculum of each bacterial load (10^5^, 10^6^, and 10^7^ CFU) was transferred into the bags with a sterile syringe (per triplicate per strain). Subsequently, five aspirations and five plasma returns ensured the inoculation of the entire bacterial load into the bag. The bags were hermetically sealed, homogenized, and immediately subjected to a sample with 3 mL of plasma to verify arterial contamination by automated microbiological culture in BD BACTEC^TM^ Plus Aerobic/F (Franklin Lakes, NJ, USA) blood culture bottles. Photoinactivation assays were performed by using the MIRASOL^®^ system (TERUMO, Somerset, NJ, USA) prior to the addition of thirty-five milliliters of riboflavin solution (500 μM) to the plasma bags according to the manufacturer’s instructions. The volume of each plasma bag and the total dose (in Joules) of UV light were registered in a database for subsequent analysis. The general working scheme of the photoinactivation of contaminated plasma bags is shown in Figure 1.

### 2.5. Culture of Surviving Bacteria after Photoinactivation

After photoinactivation, the plasma bags were subjected to the cultivation of surviving bacteria by an automated method. For this purpose, three milliliters of each plasma bag were inoculated into BD BACTEC^TM^ Plus Aerobic/F blood culture bottles. After a seven-day incubation period, samples that exhibited a positive signal of contamination were included for subsequent tests (bacterial isolation and genetic identification by PCR). Growth curves before and after photoinactivation assays were analyzed to detect the initial signal of bacterial growth (*log* phase). With this information, Logarithmic Reduction Values (LRVs) were calculated using the following equation:

L = Log10 (A) − Log10 (B),

where L is the LRV, A is the number of viable bacteria before treatment, and B is the number of viable bacteria after treatment (*log* phase lag time in the growth curves). Additionally, the Percent Reduction Values (PRVs) were calculated by using the following equation:

P = [(1 − 10^−L^) (100)],

where P is the PRV, and L is the LRV.

All the data were statistically analyzed after and before MIRASOL^®^ inactivation using Microsoft Excel 2022 (Microsoft Corporation, Redmond, WA, USA) and the Prism software package (GraphPad version 8.0).

### 2.6. Molecular Identification of Survival Bacteria after Photoinactivation

To identify the possible contamination during the manipulation of plasma post-photoinactivation, all bacterial strains recovered were isolated in solid media (LB-agar) and identified by *16S rRNA* gene sequencing obtained by PCR assays. Genomic DNA was extracted using the QIAamp DNA Mini QIAcube Kit (QIAGEN, Hilden, Germany). The reactions were performed in a Touchgene Gradient thermal cycler FTGRAD2D (TECHNE DUXFORT, Cambridge, UK) by using MasterMix PCR 1 × (Roche Diagnostics, Hilden, Germany), 200 pmol of each primer and 200 ng of template DNA. *16S rRNA* gene (V1 to V9 region) PCR was carried out with the universal primers 27F (5′-AGA GTT TGA TCM TGG CTC AG-3′) and 1492R (5′-TAC GGY TAC CTT GTT GTT ACG ACT T-3′) in accordance with the conditions recommended by De Santis et al. (2007) [19].

Amplicons were analyzed on horizontal 1% agarose gels using 1× Tris-Acetate-EDTA buffer and were purified using the Illustra GFX^TM^ PCR-DNA purification kit (General Electric, Life Sciences, Chalfont St. Giles, UK). The identity of amplicons (*16S rRNA* gene) was determined by sequencing performed by Instituto de Biología-Universidad Nacional Autonóma de México (IB-UNAM) using an ABI PRISM^®^ 310 Genetic Analyzer sequencer (Applied Biosystems, Foster City, CA, USA). Sequences were compared with the nucleotide sequence database (GenBank) by means of the BlastX algorithm (http://blast.ncbi.nlm.nih.gov, accessed on 1 February 2024) using strict filter parameters with more than 99% nucleotide homology and at least 80% query coverage.

## 3. Results

### 3.1. Photoinactivation and Its Impact on Coagulation Pathways

**Figure 2:**
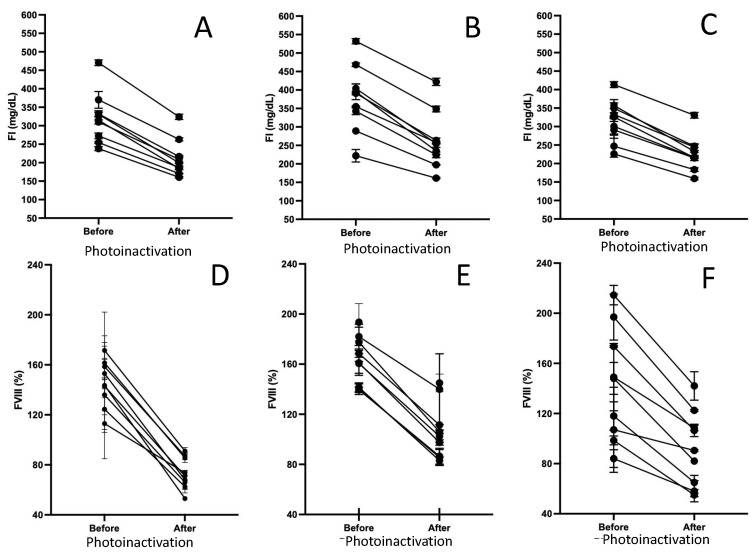
Graphical representation of the behavior of the FVIII and FI factors before and after bacterial photoinactivation (classified by bacterial strain tested). (**A**,**D**) *E. cloacea* 087, (**B**,**E**) *Klebsiella ozaenae* 008, and (**C**,**F**) *Staphylococcus epidermidis* 6(01).

The general conditions of the plasma bags undergoing photoinactivation were as follows: final volume: 197.2 ± 22.4 mL, and total dose: 1229 ± 159 Joules. The evaluation of FVIII and FI revealed the following: the activity quantification of FVIII before and after bacterial photoinactivation showed average values of 154.8 and 93.4%, respectively, which represents an average percentage of net residual activity of 61%. Alternatively, the quantification of FI before and after photoinactivation showed average values of 338.4 mg/dL and 216 mg/dL, respectively. This represents an average net residual concentration of 69% compared to the initial average concentration. No difference was identified in the behavior of the FVIII and FI factors with each of the concentrations of the bacterial strains tested. The graphical representation of the behavior of the FVIII and FI factors before and after bacterial photoinactivation (classified by bacterial strain tested) is shown in Figure 2.

### 3.2. Bacterial Load Influences Photoinactivation

**Figure 3:**
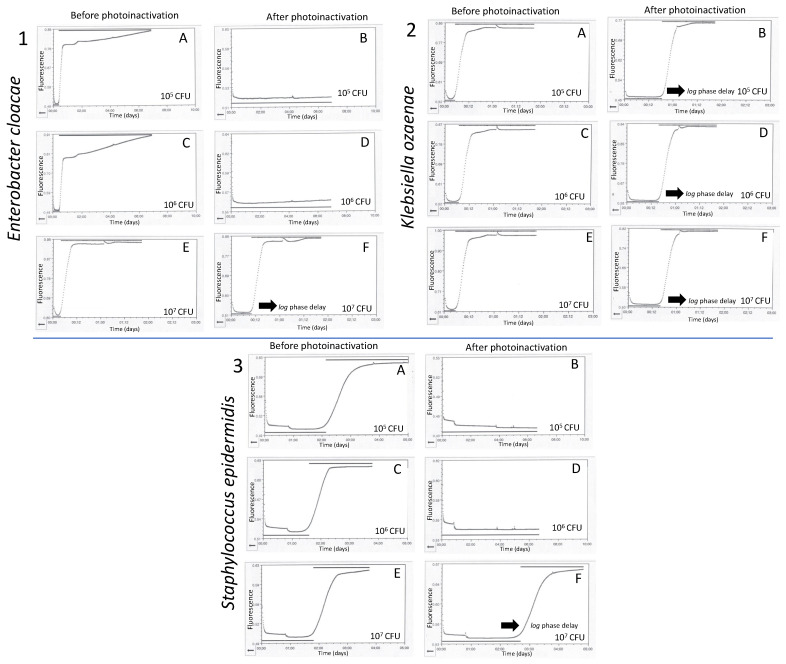
Bacterial growth curves before and after photoinactivation assays in plasma bags. **1**(**A**–**F**) *Enterobacter cloacae* 087, **2**(**A**–**F**) *Klebsiella ozaenae* 008, and **3**(**A**–**F**) *Staphylococcus epidermidis* 6(01).

The results of the photoinactivation tests revealed that *E. cloacea* 087 and *S. epidermidis* 6(01) were completely reduced after photoinactivation assays at the first two bacterial loads tested (10^5^ and 10^6^ CFU), with LRVs of 5 and 6 with a PRV of 100% (Table 2). In contrast, when subjected to photoinactivation of 10^7^ CFU of these microorganisms, LRV and PRV of 0.46 ± 0.06 and 65.3 ± 5.3%, respectively, for *E. cloacea* 087 and 0.20 ± 0.05 and 36.1 ± 7.8 for *S. epidermidis* 6(01), respectively, were achieved. Interestingly, *K. ozaenae* 008 achieved LRVs and PRVs of 0.43 ± 0.11/62.3 ± 8.9, 0.51 ± 0.37/62.2 ± 23.94, and 0.42 ± 0.1/61 ± 9.7 for 10^5^, 10^6^, and 10^7^ CFU, respectively. The average times for residual bacterial contamination detection (after photoinactivation) in BD BACTEC^TM^ Plus Aerobic/F system were 9.88 ± 0.66 h for *E. cloacae* 087 (10^7^ CFU), 10.07 ± 0.5 h (10^5^ CFU), 13.9 ± 2.65 h (10^6^ CFU),14.6 ± 2.48 for *K. ozaenae* 008, and 58.5 ± 0.9 h (10^7^ CFU) for *S. epidermidis* 6(01) (Figure 3).

Table 2 shows the microbiological conditions before and after photoinactivation in plasma bags used in this study. Finally, PCR tests against the isolates recovered after the photoinactivation assays (*16S rRNA* gene sequencing) showed 100% similarity and genetic identity with the strains used as bacterial contaminants. Therefore, it is concluded that there were no cross-contamination events after photochemical reduction.

## 4. Discussion

Since Mexico lacks a national hemovigilance system, there are no reports on the identification, tracking, and follow-up of infections associated with blood transfusion and its components. Therefore, increasing blood safety for the recipient is one of the most important axes in Mexican transfusion medicine. Blood banks have added technological advances to increase blood safety, and among them are the photoinactivation systems, also called pathogen reduction systems. Although there is sufficient information in the scientific literature on their effectiveness, highly relevant variables that could influence the efficiency of these inactivation systems have been omitted. These factors are related to the genetic background of the microorganisms subjected to photoinactivation. Therefore, in the present study, we investigated the potential use of an automated hemocomponent inactivation system considering variables that, to our knowledge, had not been explored in photoinactivation assays, with the phenotype and resistance genotype of bacteria isolated from the skin of blood donors. Consequently, the microbiological models used in the present study were chosen under a rigorous biofilm formation screening, which provides intrinsic resistance to povidone iodine and chlorhexidine gluconate/isopropyl alcohol, antiseptics used in blood banks. Conversely, the selection of microorganisms that were endowed with antimicrobial resistance phenotypes and virulence by the production of various exoenzymes considered potential virulence factors (Table 1) [10].

The results of these tests showed that the effectiveness of the photoinactivation system is dependent on two relevant aspects: the type of test microorganism and the microbiological load subjected to reduction. In the case of *E. cloacae* 087, a bacterium that has been recognized as a pathogen related to post-transfusion infectious processes, it was reduced to 100% when logarithmic concentrations of 5 (10^5^ CFU) and 6 (10^6^ CFU) were used. In contrast, when ten times higher concentrations were used, the average PRV was reduced to 65.33%. Similar to our results, a previous study has demonstrated a 100% reduction in *E. cloacae* PEI-B-P-43 at concentrations of 10^6^ CFU, employing a photoinactivation system that presents the same principle as the one used in the present work [20]. An alternative approach is the photoinactivation study reported by Goodrich et al. (2009), which allows speculation on the possible role of the genetic background of the microorganisms tested [21]. In this work of bacterial photoinactivation of platelet concentrates, the same microorganism (*E. cloacae*) was used, but it belongs to a reference culture collection (ATCC^®^ 29005). The results showed that the photoinactivation system had an efficiency of only 67% when using significantly lower inoculum than those used in this work (between 20 and 100 CFU). It is important to mention that one of the characteristics of this microorganism is its resistance to multiple antibiotics (amoxicillin-clavulanic acid, ampicillin, cefazolin, and cefoxitin) and because of its isolation origin (spinal fluid), the presence of a potential virulent background that provides protection to photoinactivation is inferred [22].

Considering these characteristics, we believe that they could be involved in the resistance to photoinactivation and would explain the results reported by Goodrich et al. [21]. From the above, we can conclude that, for our photoinactivation assays, the efficiency of this system is dependent on the load tested and the type of microorganism. Another Gram-negative microorganism isolated from the skin of blood donors and used in the photoinactivation assays was *K. ozaenae*, a bacterium widely related to infectious processes in immunosuppressed patients such as rhinitis, complicated pneumonia, bacteremia, urinary tract infections, among others [23,24,25]. This bacterium could not be reduced to 100% with the concentrations tested, and heterogeneity was observed in the experimental replicates, which was reflected in the standard deviations of the LRV and PRV (Table 2). The genus *Klebsiella* has historically been recognized as one of the main Gram-negative biofilm-forming microorganisms and, in some cases, has been categorized with a hypermucoviscous phenotype and genotype due to its exacerbated capacity for exopolysaccharide synthesis involved in virulence and bacterial adherence [26]. From the aforementioned, it is reasonable to infer that the properties described in Table 1 in *K. ozaenae* 008 are related to tolerance to photoinactivation. The significance of this microorganism as a causative agent of post-transfusion infections is such that comprehensive studies have been conducted to investigate its photoinactivation in hemocomponents. Alabdullatif et al. (2021) evaluated the effectiveness of the same inactivation system reported in this work, using different concentrations of *K. pneumoniae* of clinical and reference origin [27]. The results revealed intrinsic resistance of *K. pneumoniae* 1644802 to photoinactivation. The authors speculated the possible role of the origin of the test strain, as well as its high proliferation capacity in hemocomponents, which prevented its photoinactivation. There are few studies that have discussed the importance of the biofilm-forming capacity of bacteria to evade the reducing activity of riboflavin, such as the case of Lu and Fung (2020), who have shown their concern about the limitation of inactivation systems against bacteria that have the ability to form biofilms or bacterial aggregates in the collection bags, since these characteristics could limit the entry of riboflavin to the target of action (DNA), due to the modification of permeability by the presence of exopolysaccharides [28]. Therefore, we speculate that this phenotype of mature biofilm formation in the isolates tested in this work directly impacts the photoinactivating activity by reducing its permeability and, consequently, the entry of riboflavin.

In contrast, it could be assumed that among the weaknesses of this work is the use of strains that are not part of the WHO International Repository Platelet Transfusion Relevant Reference strains (WHO-IRPTR), validated for pathogen inactivation assays in blood and blood components [17]. Nevertheless, our research indicates that the use of strains isolated from the skin represents a strength since the detected phenotypic and genotypic characteristics described above provide new information on the spectrum of photochemical reduction systems (Table 1), which, to our knowledge, has not been considered in any other work. Finally, the final bacterial model tested in this study was *E. epidermidis*, a bacterium that has been globally associated with contamination events involving blood and its components. In previous studies conducted within our research group, *E. epidermidis* has been linked to inadequate antisepsis of the ulnar area of blood donors [10,29]. This microorganism is regarded as innocuous due to its presence within the skin microbiota. However, in other circumstances, it can function as a pathogen in immunocompromised patients, such as those who have received blood or components [30,31]. Kou et al. 2015 have recognized the importance of the biofilm-forming phenotype in this microorganism as a causative agent of bacteremia in a splenectomized oncology patient when transfused with a contaminated blood component [32].

The results of the photochemical reduction of this microorganism showed a behavior similar to *E. cloacae* 087, with load dependence. This phenomenon was observed with bacterial concentrations of 10^7^ CFU with PRVs of 36.08% (Table 2). In a previous study by our working group, using *S. epidermidis* PEI-B-P-06, WHO-IRPTR reference strain, we showed the sensitivity of this microorganism to photoinactivation up to 10^6^ CFU, with a PRV of 99.99%, which contrasts with our results, since using the same bacterial concentration of *S. epidermidis* 6(01) reduction was achieved at 100%. With this observation, we can conclude that, particularly for this microorganism, even when it is endowed with phenotypic and genotypic characteristics of protection, the system used in this work manages to reduce the microorganism in bacterial densities of up to 10^6^ CFU. Then again, previous studies have shown that in UV light and riboflavin, in addition to inducing bacterial inactivation, the activity of coagulation factors in plasma and other hemocomponents is decreased [16,33]. This reduction is given by the nature of the factors since, being labile molecules, their activity is modified (downward) from the moment the hemocomponents are stored at freezing temperatures and after photochemical treatment. Therefore, to know the degree of modification of the activity of the coagulation factors after bacterial reduction with riboflavin, two key factors in secondary hemostasis, FVIII and FI, were evaluated; their measurement was considered within the parameters for quality control of plasmas and cryoprecipitates after thawing. As shown in Figure 2, the results of residual net activity of 61 and 69% for FVIII and FI, respectively, show similarity with those reported by Hornsey et al. (2009), which show residual percentages of activity for FVIII of 68.5 ± 3.3% and IF of 78.8 ± 4.5% [33].

Other studies have indicated that the integrity of other factors, such as FV, FVII, and FXII, were not significantly affected even when the plasmas were frozen for up to two years, which could allow us to speculate a behavior similar to our assays [34]. These authors have mentioned that even when there is a reduction in clotting factor activity, it is unlikely to have a negative impact on the recipients. It is important to mention that the national regulation given by NOM-253-SSA1-2012 refers to an expected loss of 15% maximum when plasma is subjected to an inactivation process, contrasting with the results obtained in the present work [5]. However, with the results obtained by previous studies, we consider that we are within the expected values for these coagulation factors after photochemical reduction. Further clinical research is needed to test this hypothesis since the few clinical trials do not show conclusive findings on their effectiveness in other hemocomponents subjected to photoinactivation and on the evaluation of risk associated with transfusion of blood and photochemically reduced hemocomponents [35,36]. The evidence presented indicates that the evaluation of coagulation factors should be a fundamental activity in pathogen inactivation assays. This is because, even when their reducing effectiveness on emerging and re-emerging pathogens such as SARS-CoV-2 and monkeypox virus, respectively, is demonstrated, the evaluation of the activity of the fundamental purpose of transfusion of safe hemocomponents, namely the restoration of hemostasis, has been neglected [15,37,38,39].

## 5. Conclusions

As evidenced by the findings presented in this study, photoinactivation, such as MIRASOL^®^, represents a crucial approach to ensuring the safety of blood and blood components for recipients in countries where blood banks lack the necessary detection technologies for pathogens, including those that are emerging and could go unnoticed in the screening of blood donors. Finally, this approach not only offers an effective way to mitigate the risk of pathogen transmission but also shows new information about the spectrum of these technologies by reducing pathogens with phenotypic and genotypic characteristics that allow them to resist adverse conditions such as UV light and anti-DNA agents.

## Figures and Tables

**Table 1:** Bacterial strains used in this work.

Bacterial Strain	Source	Phenotypic and Genetic Characteristics	Reference
*Enterobacter cloacae* 087	Skin	Gram-negative, povidone iodine-resistant (10%), chlorhexidine gluconate/isopropyl alcohol (3%/70%), strong biofilm former, *csgA*^+^ and *csgD*^+^ genes, a^+^ hemolysis, amylase^+^, TZP^R^, ETP^I^, IPM^I^.	[10]
*Klebsiella ozaenae* 008		Gram-negative, povidone iodine-resistant (10%), chlorhexidine gluconate/isopropyl alcohol (3%/70%), strong biofilm former, a^+^ hemolysis, amylase^+^ SAM^R^, TZP^R^, CRO^R^, AN^R^, AK^R^, IPM^I^, FEP^R^.	
*Staphylooccus epidermidis* 6(01)		Gram-positive, povidone iodine-resistant, strong biofilm former, *icaA*^+^, *icaD*^+^ genes, lipase^+^, amylase^+^, protease^+^, E^R^.	

^R^: Resistance, ^I^: Intermediate, ^+^: Producer. TZP: Piperacillin/Tazobactam, SAM: Ampicillin/Sulbactam, CRO: Ceftriaxone. AN: Amikacin, FEP: Cefepime, ETP: Ertapenem, IPM: Imipenem, E: Erythromycin.

**Table 2:** Microbiological conditions of plasma bags before versus after photoinactivation and Logarithmic Reduction Values (LRVs) and Percent Reduction Values (PRVs).

Bacterial Strain	CFU Tested	Plasma Bag	Volumen (mL) per Bag	Dose (Joules)	Bacterial Growth *		Photoinactivation	
					Before Reduction	After Reduction	LRV **	PRV ***
*Enterobacter cloacea* 087	10^5^	1	174	1085	+	−	5	100
	10^5^	2	190	1184	+	−	5	100
	10^5^	3	192	1197	+	−	5	100
	10^6^	4	234	1458	+	−	6	100
	10^6^	5	207	1290	+	−	6	100
	10^6^	6	192	1197	+	−	6	100
	10^7^	7	218	1359	+	+	0.39	59.26
	10^7^	8	215	1340	+	+	0.49	67.64
	10^7^	9	167	1041	+	+	0.51	69.09
*Klebsiella ozaenae* 008	10^5^	10	186	1159	+	+	0.35	55.33
	10^5^	11	169	1053	+	+	0.39	59.26
	10^5^	12	188	1172	+	+	0.56	72.45
	10^6^	13	238	1483	+	+	0.23	41.11
	10^6^	14	188	1172	+	+	0.37	57.34
	10^6^	15	171	1066	+	+	0.93	88.25
	10^7^	16	223	1390	+	+	0.30	49.88
	10^7^	17	197	1228	+	+	0.48	66.88
	10^7^	18	195	1215	+	+	0.47	66.11
*Staphylococcus epidermidis* 6(01)	10^5^	19	180	1122	+	−	5	100
	10^5^	20	167	1041	+	−	5	100
	10^5^	21	231	1440	+	−	5	100
	10^6^	22	227	1415	+	−	6	100
	10^6^	23	167	1041	+	−	6	100
	10^6^	24	180	1122	+	−	6	100
	10^7^	25	199	1240	+	+	0.17	32.39
	10^7^	26	208	1296	+	+	0.16	30.81
	10^7^	27	223	1390	+	+	0.26	45.05

* By BD BACTEC^TM^ Plus Aerobic/F, ** Logarithmic Reduction Value, *** Percent Reduction Value.

## Data Availability

Plasma Photoinactivation of Bacterial Isolated from Blood Donors Skin: Potential of Security Barrier in Transfusional Therapy_Yanet Ventura-Enríquez et al., 2024_DOI:10.17632/276dwzdd5x.1 (https://data.mendeley.com/datasets/276dwzdd5x/1, accessed on 1 February 2024).
